# Association of dysmorphic intratumoral vessel with high lung shunt fraction in patients with hepatocellular carcinoma

**DOI:** 10.1038/s41598-022-18697-5

**Published:** 2022-08-21

**Authors:** Tae Won Choi, Ijin Joo, Hyo-Cheol Kim

**Affiliations:** Department of Radiology, Seoul National University Hospital, Seoul, 03080 Republic of Korea

**Keywords:** Hepatocellular carcinoma, Hepatocellular carcinoma, Cancer imaging

## Abstract

To evaluate the role of dysmorphic intratumoral vessels as imaging marker for the prediction of high lung shunt fraction (LSF) in patients with hepatocellular carcinoma (HCC). This retrospective study included 403 patients with HCC who underwent a planning arteriography for transarterial radioembolization with administration of ^99m^Tc-macroaggregated albumin to calculate LSF. The LSF was measured by using planar body scans. Two radiologists evaluated the pre-treatment contrast-enhanced CT findings, including tumor number, size, margin, distribution, tumor burden, portal and hepatic vein invasion, early hepatic vein enhancement, and dysmorphic intratumoral vessels. The logistic regression analysis was performed to determine significant predictors for high LSF > 20%. Using the identified predictors, diagnostic criteria for high LSF were proposed. Among 403 patients, 52 (13%) patients had high LSF > 20%, and dysmorphic tumor vessels were present in 115 (28.5%) patients. Predictors for LSF > 20% were tumor size > 11 cm, hepatic vein invasion, early hepatic vein enhancement, and dysmorphic intratumoral vessel. If the patient had three or more of the four predictors for LSF > 20% on imaging, the accuracy and specificity for diagnosing LSF > 20% were 88.8% and 96.3% respectively. Dysmorphic intratumoral vessel in HCC is an imaging marker suggesting a high LSF, which may be applicable to treatment modification or patient exclusion for radioembolization with combined interpretation of tumor size and hepatic vein abnormality.

## Introduction

Transarterial radioembolization (TARE) is a transcatheter intra-arterial therapy that selectively delivers microspheres that contain radioisotope yttrium-90 (^90^Y) to the target tumors. It is gaining popularity as a viable alternative to conventional transarterial chemoembolization (cTACE), which is a standard treatment modality for hepatocellular carcinoma (HCC) at the Barcelona Clinic Liver Cancer stage B^1,2^. Although additional randomized controlled trials are required to determine the detailed roles of TARE in comparison with cTACE in managing HCC patients, TARE was reported to exhibit a longer progression-free survival and a lower rate of adverse events^1,3–7^. Recently, DOSISPHERE-01 trial showed promising results that the outcome of TARE can be further improved by using personalized dosimetry^8^.

However, a fraction of microspheres injected into the hepatic artery was known to pass through abnormal intrahepatic shunt and reach the pulmonary microvascular bed, potentially causing fatal radiation pneumonitis^9,10^. Therefore, the lung shunt fraction (LSF) assessed by ^99m^Technetium macroaggregated albumin (^99m^Tc-MAA) scans is important in the patient selection and determination of the ^90^Y dosage^3^. For resin microspheres, TARE is contraindicated in patients with high LSF > 20% and dose reduction is recommended in patients with LSF > 10%^3,11^. For glass microspheres, TARE is contraindicated if the expected lung dose is > 30 Gy per session or > 50 Gy in life, which is usually associated with high LSF^3,11^.

Previous studies have reported imaging predictors of high LSF, including hepatic vein invasion, early opacification of the hepatic vein, infiltrative tumor, tumor burden > 50%, main portal vein invasion, and arterioportal shunting^12,13^. According to the authors’ experiences, dysmorphic intratumoral vessels frequently observed in large HCCs on arterial phase computed tomography (CT) images may also be associated with high LSF. To the best of our knowledge, there are no other studies who reported associations between dysmorphic intratumoral vessels and high LSF. The purpose of the present study is to evaluate the role of dysmorphic intratumoral vessel as a novel imaging marker for the prediction of high LSF in patients with HCC.

## Materials and methods

This retrospective study was approved and the need for informed consent was waived considering the retrospective study nature by the Seoul National University Hospital Institutional Review Board (2101-014-1186). The study and its procedures complied with the tenets of the Declaration of Helsinki.

### Patients

The authors searched the electronic medical records of our hospital and identified patients who met all of the following inclusion criteria: patients (a) with HCC, (b) who underwent preprocedural contrast-enhanced CT, and (c) patients who underwent planning arteriography for TARE procedures between January 2012 and May 2021. As a result, a total of 403 patients (mean age, 63 ± 12 years [range, 24–92 years]), which comprised 343 men (mean age, 63 ± 12 years [range, 25–89 years]) and 60 women (mean age, 66 ± 14 years [range, 24–92 years]) were included in the present study.

### Liver CT protocol

Preprocedural CT examinations were performed with the use of various multidetector row CT scanners and following imaging parameters: peak voltage of 100–120 kVp, tube current of 150–250 mAs, slice thickness of 2.5–3.0 mm, and reconstruction interval of 2.0–3.0 mm^14^. The liver CT protocol at the authors’ institution consisted of four phases: precontrast, hepatic arterial, portal venous, and delayed phase images. After the precontrast scans were conducted, nonionic contrast medium (1.6 mL/kg) was injected via intravenous route with the use of an automatic power injector. The timing of the scanning was determined using the bolus tracking technique. The hepatic arterial and portal venous phase scans started at 17–19 s and 45–50 s, respectively, after attenuation in the descending thoracic aorta reached 100 Hounsfield units.

### Planning arteriography and calculation of the lung shunt fraction

Celiac and superior mesenteric arteriography was routinely performed, and cone-beam CT was obtained at the proper/common hepatic artery or at an equivalent anatomical location. ^99m^Tc-MAA (185 MBq) was injected at the right or left lobar hepatic arteries which supplied the primary target tumor. After the injection of ^99m^Tc-MAA, the patients were transported from the interventional suite to the nuclear medicine department. Whole body planar scan was performed within 1 h after the injection of ^99m^Tc-MAA. Anterior and posterior images were obtained simultaneously using a dual-head gamma camera (Discovery NM/CT 670, GE Healthcare), equipped with low-energy high-resolution parallel collimators. The scan was performed using a table-speed setting (18 cm/min), and images were obtained with the energy window centered at 140 keV (± 15%), and matrix size of 1024 × 256.The LSF was determined that total lung counts was divided by total lung and liver counts using both anterior and posterior planar body scans.

### Image analysis

Two radiologists (T.W.C. with 1 year of experience in interventional oncology, I.J. with 10 years of experience in liver imaging) reviewed CT images in consensus on a picture-archiving and communication system (Infinitt PACS Viewer; Infinitt, Seoul, Republic of Korea) and determined the following image findings: number of tumors (single vs. multiple), tumor margin (circumscribed vs. infiltrative), tumor distribution (unilobar vs. bilobar), tumor burden (> 50% vs. ≤ 50% of the total liver volume), portal vein invasion, hepatic vein invasion, early hepatic vein enhancement, and dysmorphic intratumoral vessel (presence vs. absence;). The presence of the early hepatic vein enhancement was determined in comparison with the other unaffected hepatic veins on the arterial phase image^9,13^. Dysmorphic intratumoral vessels were defined as linear or saccular structures in the tumor which exhibited enhancement at a degree which was similar to that of arteries on arterial phase CT image, which is not hepatic artery branch itself^14^. In this study, dysmorphic intratumoral vessels were regarded as present when their diameter is over 3 mm. Disagreement on image findings was resolved in consensus in additional image review sessions with senior radiologist (H.C.K. with 14 years of experience in interventional oncology).

### Statistical analysis

The receiver operating characteristic curve analysis was performed to determine the optimal cutoff values of the tumor size for the determination of high LSF > 20%. The values which maximized the Youden’s index (sensitivity + specificity − 1) were used as the cutoff values.

Descriptive statistics were performed and comparisons between groups were performed with the use of the Pearson’s chi-square test. Thereafter, logistic regression analysis was performed to determine predictors for high LSF > 20%. Initially, univariate analysis was performed for each variable. Only variables with P values < 0.05 were included in the multivariate analysis. The forward conditional method was used for the analysis. The variance inflation factors (VIFs) were calculated to confirm that there was no significant multicolinearity between variables included in the final model. VIF values > 10 indicated the presence of significant multicollinearity. With the use of the independent predictors identified in logistic regression analysis, diagnostic criteria for high LSF were proposed and sensitivity, specificity, and accuracy were calculated.

All statistical analyses were performed with the use of SPSS (version 26.0, IBM, Armonk, NY, USA) and P values < 0.05 were considered to indicate statistically significant differences.

## Results

Among 403 patients, 304 patients (75.4%) underwent TARE procedure. Glass microspheres (TheraSphere, Boston Scientific, Natick, MA, USA) were used in 277 patients (91.1%) and resin microspheres (SIR-Spheres, Sirtex Medical, Lane Cove, Australia) were used in 27 patients (8.9%).

Patient characteristics and the prevalence of high LSF > 20% are presented in Table 1. Of the 403 patients, 52 (12.9%) had high LSF > 20%, among whom 47 patients underwent cTACE and the other 5 patients underwent TARE with reduced dose.

**Table 1:** Association between patients’ characteristics and lung shunt fraction.

	Lung shunt fraction > 20%	P
**Age**		
> 60	10.9% (27/247)	0.137
≤ 60	16.0% (25/156)	
**Sex**		
Male	13.7% (47/343)	0.252
Female	8.3% (5/60)	
**Tumor size**		
≤ 11 cm	4.9% (13/265)	< 0.001
> 11 cm	28.3% (39/138)	
**Tumor number**		
Single	13.1% (28/214)	0.908
Multiple	12.7% (24/189)	
**Margin**		
Circumscribed	14.7% (46/312)	0.041
Infiltrative	6.6% (6/91)	
**Tumor burden**		
Unilobar	11.9% (28/235)	0.484
Bilobar	14.3% (24/168)	
> 50%	25.0% (20/80)	< 0.001
≤ 50%	9.9% (32/323)	
**PV invasion**		
Present	10.8% (15/139)	0.359
Absent	14.0% (37/264)	
**HV invasion**		
Present	37.5% (15/40)	< 0.001
Absent	10.2% (37/363)	
**Early HV enhancement**		
Present	33.3% (18/54)	< 0.001
Absent	9.7% (34/349)	
**Dysmorphic intratumoral vessel**		
Present	36.5% (42/115)	< 0.001
Absent	3.5% (10/288)	

^†^P-values were calculated using the Pearson's chi-square test.

**Figure 1:**
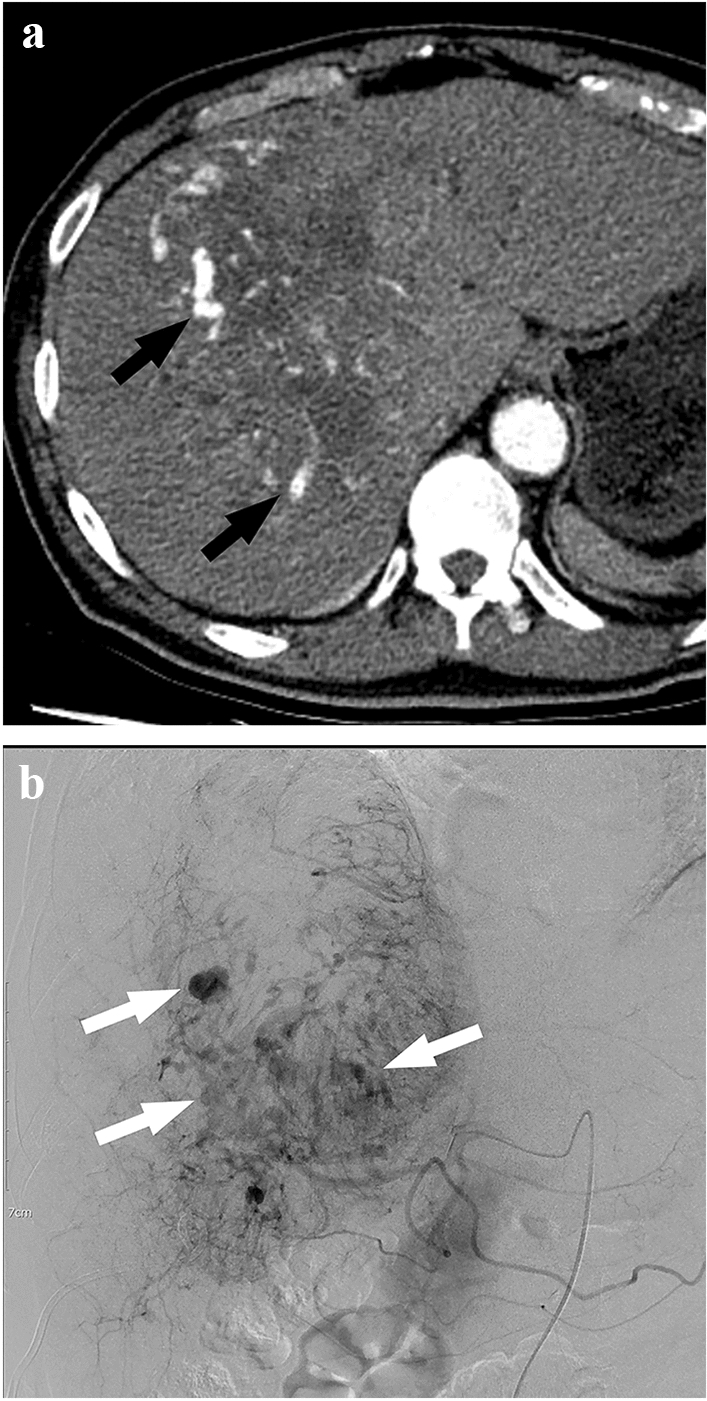
A 67 year old man with hepatocellular carcinoma (size equal to 16 cm). (**a**,**b**) Arterial phase CT images (**a**) and hepatic arteriography (**b**) revealed multifocal dysmorphic intratumoral vessels with sizes up to 9 mm (arrow). The lung-shunt fraction was 44.74%.

Dysmorphic intratumoral vessels were present in 115 (28.5%) of 403 patients (Fig. 1). Dysmorphic intratumoral vessels were found in 35.6% of circumscribed tumors (111/312) and in 4.4% of infiltrative tumors (4/91). They were also identified in 1.5% (1/68), 13.8% (22/160), and 52.6% (92/175) patients with tumor sizes < 5 cm, 5–10 cm, and > 10 cm, respectively.

The optimal cutoff values of tumor size for the prediction of LSF > 20% were 11 cm. The results of logistic regression analysis for the identification of independent predictors for high LSF > 20% are listed in Table 2. Independent predictors for high LSF > 20% were tumor sizes > 11 cm, hepatic vein invasion, early hepatic vein enhancement, and dysmorphic intratumoral vessels. The diagnostic criteria for the prediction of high LSF were made with the use of independent predictors for high LSF described above (Table 3). If the patient had three or more of the four predictors for LSF > 20% on imaging, the accuracy and specificity for diagnosing LSF > 20% were 88.8% and 96.3% respectively.

**Table 2:** Predictors for lung shunt fraction higher than 20%.

Risk factors	Univariate analysis			Multivariate analysis		
	P	OR	95% CI	P	OR	95% CI
Age > 60	0.139	0.643	0.358–1.155			
Male	0.258	1.747	0.665–4.588			
Tumor size > 11 cm	< 0.001	7.636	3.910–14.914	0.009	2.793	1.291–6.042
Multiple tumors	0.906	0.966	0.539–1.733			
Circumscribed margin	0.047	2.450	1.011–5.937			
Bilobar disease	0.484	1.232	0.686–2.212			
Tumor burden > 50%	< 0.001	3.031	1.624–5.657			
Portal vein invasion	0.360	0.742	0.392–1.406			
Hepatic vein invasion	< 0.001	5.286	2.561–10.913	0.004	3.681	1.509–8.982
Early hepatic vein enhancement	< 0.001	4.632	2.377–9.028	0.010	2.809	1.282–6.157
Dysmorphic intratumoral vessel	< 0.001	15.995	7.660–33.398	< 0.001	9.022	4.037–20.163

**Table 3:** Sensitivity and specificity of diagnostic criteria for predicting high lung shunt fraction higher than 20%.

Diagnostic criteria for high lung shunt fraction > 20%	Sensitivity (%)	Specificity (%)	Accuracy (%)
High risk imaging finding^a^ ≥ 1	90.4	55.8	60.3
High risk imaging finding ≥ 2	80.8	81.5	81.4
High risk imaging finding ≥ 3	38.5	96.3	88.8
All four high risk imaging findings	9.6	100.0	88.3

^a^Tumor size > 11 cm, hepatic vein invasion, early hepatic vein enhancement, and dysmorphic intratumoral vessel.

## Discussion

**Figure 2:**
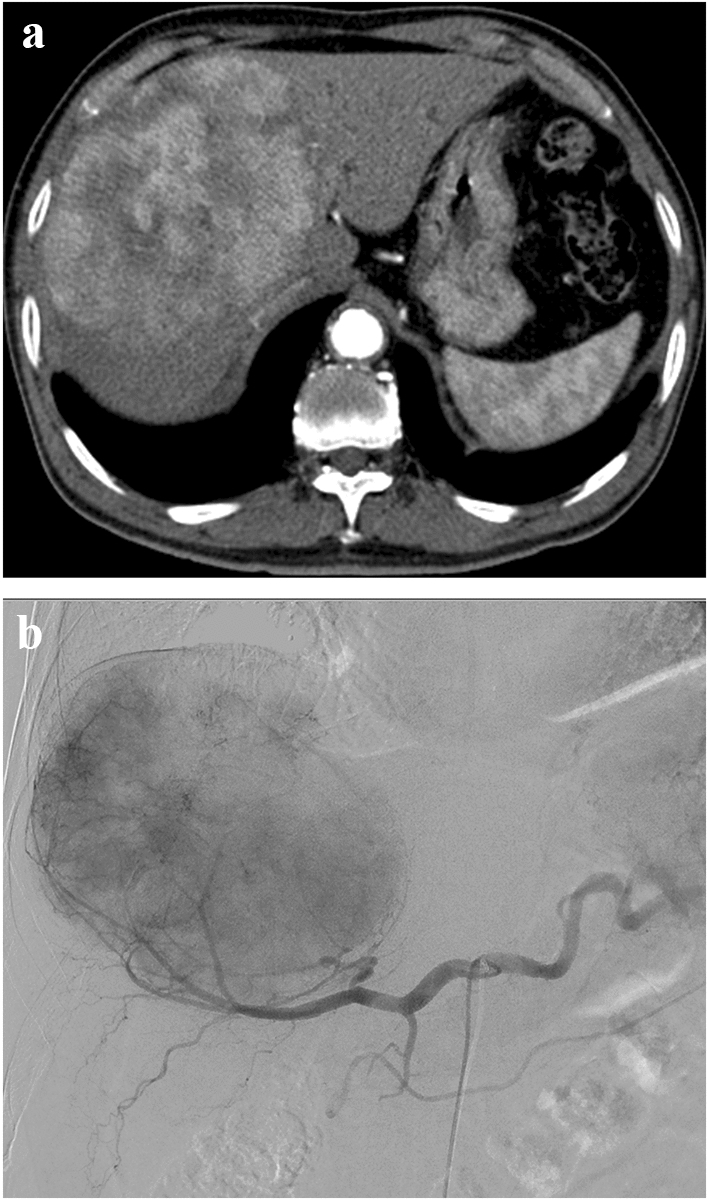
A 77 year old man with a hepatocellular carcinoma (size equal to 15 cm). (**a**,**b**) No dysmorphic intratumoral vessels were found on preprocedural liver computed tomography (CT) scans (**a**), and planning hepatic arteriography (**b**). In addition, there was no hepatic vein invasion or early hepatic vein enhancement on CT. The lung-shunt fraction was 0.79%.

In this study, dysmorphic intratumoral vessels are commonly present in large circumscribed tumors. According to authors’ experiences, whereas tumors without dysmorphic intratumoral vessels have commonly low LSF (Fig. 2), tumors with dysmorphic intratumoral vessels seems to frequently have high LSF (Fig. 1).

The present study demonstrated that the presence of dysmorphic intratumoral vessel was independent predictor for high LSF. In the literature, the presence and aneurysmal dilatation of dysmorphic intratumoral vessels have been reported to be more frequently observed in poorly differentiated HCC compared with well-differentiated HCC^15^. Dysmorphic intratumoral vessels are different from the hepatic artery branch inside the tumor showing infiltrative growth^16^. The dysmorphic intratumoral vessels were also different from the “vascular lake” which appeared after the embolization^16,17^. In the clinicopathologic analysis conducted by Yamanaka et al., the “condensed pooling” on angiography that corresponds to the dysmorphic intratumoral vessel in the present study was confirmed on histopathological analyses to be blood-filled cavities partly lined by the endothelium, which was a mixture of dilated tumor vessels and necrotic foci^19^. The possible explanations for the development of the dysmorphic intratumoral vessel included angiogenesis, distortion of the vascular architecture, and the development of arteriovenous anastomosis^15,16,20,21^. Thus, dysmorphic intratumoral vessels may have dilated channels to the hepatic vein, thus resulting in high-LSF values.

Several studies have been published that have identified imaging predictors for high-LSF cases^9,12,13^. Gaba et al. reported that infiltrative tumors, tumor burden > 50%, portal vein invasion, and arterioportal shunting, were independent predictors for high LSF for HCC^12^. Olorunsola et al. reported the early hepatic vein opacification and hepatic vein tumor thrombus or occlusion as independent predictors for high LSF^13^. The importance of hepatic vein invasion and early hepatic vein enhancement in the prediction of high LSF was also confirmed in the present study. The prevalence of early hepatic vein enhancement on arterial phase cross-sectional imaging in the present study (13.4%, 54/403) was similar to those reported by Olorunsola et al. (12.8%, 15/117)^13^ and Bermo et al. (17.3%, 59/342)^9^. In the present study, however, infiltrative tumor, tumor burden > 50%, portal vein invasion, and arterioportal shunting, were not significant for the prediction of high LSF. Interestingly, in this study, high LSF > 20% is more frequent in the circumscribed tumor than in the infiltrative tumor. In addition, dysmorphic intratumoral vessels are rare in infiltrative tumor.

The results of the present study may have important implications as various imaging features were comprehensively evaluated in a relatively large number of HCC patients (n = 403). Although they also comprehensively analyzed various imaging features, the number of included HCC patients was relatively small in the studies conducted by Gaba et al. (n = 70)^12^ and Olorunsola et al. (n = 50)^13^. The study by Bermo et al. included a relatively large number of HCC patients (n = 282), but they evaluated only imaging findings associated with the shunt^9^.

When compared with cTACE, one disadvantage of TARE is the necessity of planning angiography and lung shunt measurement, resulting in treatment delay of 1 ~ 2 weeks. The results of the present study may provide important clinical implications altering workflow in clinical practice. Bland embolization or cTACE can reduce LSF when it was performed prior to the TARE procedure^22–24^. Thus, cTACE can be recommended as an alternative initial treatment option for HCC in patients with high LSF > 20%. If we can identify patients with high LSF using preprocedural imaging findings with sufficiently high specificity, these patients can omit planning angiography and undergo cTACE without treatment delay. In the present study, if the patient has three or more of the four predictors for LSF > 20% on imaging, the specificity for diagnosing LSF > 20% was as high as 96.3% (Table 3). Therefore, we suggest that patients showing three or more predictors for LSF > 20% may benefit when planning angiography/^99m^Tc-MAA scans are omitted, and when they undergo cTACE as an initial treatment without treatment delay. In the study population of the present study, if patients with three or four imaging predictors had been considered to have high LSF > 20%, 20 patients could have omitted the planning angiography/^99m^Tc-MAA scans.

There are limitations that should be noted. First, although we suggested certain tumor diameter values or dysmorphic intratumoral vessels as risk factors for high LSF, reproducibility issues in measurement may exist. Second, the incidence of dysmorphic intratumoral vessels may be underestimated in this study. In the present study, when the diameters of dysmorphic intratumoral vessels was over 3 mm, they were regarded as present. The cut-off value of 3 mm was arbitrarily determined to facilitate the differentiation between dysmorphic intratumoral vessels and normal hepatic arteries. Third, estimated lung dose depends on LSF as well as target tissue volume and desired absorbed dose. Thus, when tumor is larger than 11 cm, TARE can not be performed in most patients with LSF of 15 ~ 20%.

In conclusion, dysmorphic intratumoral vessel is a novel imaging predictor for high-LSF cases. Patients with high LSF values can be predicted with high accuracy and specificity using preprocedural cross-sectional imaging findings.

## Data Availability

The datasets generated or analyzed during the study are available from the corresponding author on reasonable request.
